# Author Correction: Conserved and divergent gene regulatory programs of the mammalian neocortex

**DOI:** 10.1038/s41586-023-07013-4

**Published:** 2024-01-10

**Authors:** Nathan R. Zemke, Ethan J. Armand, Wenliang Wang, Seoyeon Lee, Jingtian Zhou, Yang Eric Li, Hanqing Liu, Wei Tian, Joseph R. Nery, Rosa G. Castanon, Anna Bartlett, Julia K. Osteen, Daofeng Li, Xiaoyu Zhuo, Vincent Xu, Lei Chang, Keyi Dong, Hannah S. Indralingam, Jonathan A. Rink, Yang Xie, Michael Miller, Fenna M. Krienen, Qiangge Zhang, Naz Taskin, Jonathan Ting, Guoping Feng, Steven A. McCarroll, Edward M. Callaway, Ting Wang, Ed S. Lein, M. Margarita Behrens, Joseph R. Ecker, Bing Ren

**Affiliations:** 1 Department of Cellular and Molecular Medicine, University of California, San Diego School of Medicine, La Jolla, CA USA; 2 Center for Epigenomics, University of California, San Diego School of Medicine, La Jolla, CA USA; 3 Bioinformatics and Systems Biology Program, University of California, San Diego, La Jolla, CA USA; 4 Genomic Analysis Laboratory, The Salk Institute for Biological Studies, La Jolla, CA USA; 5 Division of Biological Sciences, University of California, San Diego, La Jolla, CA USA; 6 Computational Neurobiology Laboratory, The Salk Institute for Biological Studies, La Jolla, CA USA; 7 Department of Genetics, The Edison Family Center for Genome Sciences & Systems Biology, Washington University School of Medicine, St Louis, MO USA; 8 Princeton Neuroscience Institute, Princeton University, Princeton, NJ USA; 9 Department of Genetics, Harvard Medical School, Boston, USA; 10 Stanley Center for Psychiatric Research, Broad Institute of MIT and Harvard, Cambridge, MA USA; 11 McGovern Institute for Brain Research, Department of Brain and Cognitive Sciences, Massachusetts Institute of Technology, Cambridge, MA USA; 12 Allen Institute for Brain Science, Seattle, WA USA; 13 Systems Neurobiology Laboratories, The Salk Institute for Biological Studies, La Jolla, CA USA; 14 Department of Neurosciences, University of California San Diego, La Jolla, CA USA; 15 McDonnell Genome Institute, Washington University School of Medicine, St. Louis, MO USA; 16 Department of Neurological Surgery, University of Washington, Seattle, WA USA; 17 Howard Hughes Medical Institute, The Salk Institute for Biological Studies, La Jolla, CA USA; 18 Institute of Genomic Medicine, Moores Cancer Center, School of Medicine, University of California San Diego, La Jolla, CA USA

**Keywords:** Epigenetics, Evolutionary genetics, Epigenetics in the nervous system, Epigenomics

Correction to: *Nature* 10.1038/s41586-023-06819-6 Published online 13 December 2023

In the version of the article initially published, in Figs. 2c and d and 5e, numbers in labels had been rounded. These have been corrected to exact numbers. In Fig. 2c and d, the labels now read “Mammal level 3 (16,068)”, “Human biased ↑ (10,743)” and “Human biased ↓ (1,105)”. Labels in Fig. 5e now read “229,645 human ABC enhancer–gene pairs”, “Specific (2,750)”, “Mammal level 0 (151,109)”, “Mammal level 1 (25,476)” and “Mammal level 2 (15,143)”. These corrections have been made in the HTML and PDF versions of the article.

