# Supplementary material for: Conserved and divergent gene regulatory programs of the mammalian neocortex
Source: Nature. 2023 Dec 13;624(7991):390–402. doi: 10.1038/s41586-023-06819-6 (PMC10719095; doi:10.1038/s41586-023-06819-6)
Supplement: Supplementary file 1 — Legends for Supplementary Tables 1–34. [file 41586_2023_6819_MOESM1_ESM.pdf]

nature portfolio

https://doi.org/10.1038/s41586-023-06819-6

Supplementary information

Conserved and divergent gene regulatory programs of the mammalian neocortex

In the format provided by the authors and unedited

Legends for Supplementary Tables 1-34 (see separate zip file for tables)

Supplementary Table 1
Conservation GLS T-statistic for gene expression.
Format: Tab Separated text file

Supplementary Table 2
Pairwise differential gene expression.
Format: Excel sheet

Supplementary Table 3
Conservation and divergence annotation of human protein-coding gene expression.
Format: Tab Separated text file

Supplementary Table 4
Human ATAC peak summary.
Format: Tab Separated text file

Supplementary Table 5
Macaque ATAC peak summary.
Format: Tab Separated text file

Supplementary Table 6
Marmoset ATAC peak summary.
Format: Tab Separated text file

Supplementary Table 7
Mouse ATAC peak summary.
Format: Tab Separated text file

Supplementary Table 8
Conservation and divergence annotation of human ATAC peaks.
Format: Tab Separated text file

Supplementary Table 9
Conservation GLS T-statistics for chromatin accessibility.
Format: Tab Separated text file

Supplementary Table 10A
Human Vs macaque differential chromatin accessibility.
Format: Excel sheet

Supplementary Table 10B
Human Vs marmoset differential chromatin accessibility.
Format: Excel sheet

Supplementary Table 10C
Human Vs mouse differential chromatin accessibility.
Format: Excel sheet

Supplementary Table 10D
Macaque Vs marmoset differential chromatin accessibility.
Format: Excel sheet

Supplementary Table 10E
Macaque Vs mouse differential chromatin accessibility.
Format: Excel sheet

Supplementary Table 10F
Marmoset Vs mouse differential chromatin accessibility.
Format: Excel sheet

Supplementary Table 11
Human DMRs.
Format: Tab Separated text file

Supplementary Table 12
Macaque DMRs.
Format: Tab Separated text file

Supplementary Table 13
Marmoset DMRs.
Format: Tab Separated text file

Supplementary Table 14
Mouse DMRs.
Format: Tab Separated text file

Supplementary Table 15
Conservation of human DMRs.
Format: Tab Separated text file

Supplementary Table 16
Conservation GLS T-statistic for DMRs.
Format: Tab Separated text file

Supplementary Table 17
Human boundaries.
Format: Tab Separated text file

Supplementary Table 18
Macaque boundaries.
Format: Tab Separated text file

Supplementary Table 19
Marmoset boundaries.
Format: Tab Separated text file

Supplementary Table 20
Mouse boundaries.
Format: Tab Separated text file

Supplementary Table 21
Conservation of human boundaries.
Format: Tab Separated text file

Supplementary Table 22
Human loops.
Format: Tab Separated text file

Supplementary Table 23
Macaque loops.
Format: Tab Separated text file

Supplementary Table 24
Marmoset loops.
Format: Tab Separated text file

Supplementary Table 25
Mouse loops.
Format: Tab Separated text file

Supplementary Table 26
Conservation of human loops.
Format: Tab Separated text file

Supplementary Table 27
Human ABC.
Format: Tab Separated text file

Supplementary Table 28
Macaque ABC.
Format: Tab Separated text file

Supplementary Table 29
Marmoset ABC.
Format: Tab Separated text file

Supplementary Table 30
Mouse ABC.
Format: Tab Separated text file

Supplementary Table 31
Conservation of human ABC.
Format: Tab Separated text file

Supplementary Table 32
Conservation of H3K27ac at cCREs.
Format: Tab Separated text file

Supplementary Table 33
Human divergent enhancer-gene pairs.
Format: Tab Separated text file

Supplementary Table 34
Sample sizes for Figure 4e, 4d, 5b, 5d, Extended Data 7f.
Format: Excel sheet
