# Supplementary material for: Conserved and divergent gene regulatory programs of the mammalian neocortex
Source: Nature. 2023 Dec 13;624(7991):390–402. doi: 10.1038/s41586-023-06819-6 (PMC10719095; doi:10.1038/s41586-023-06819-6)
Supplement: Supplementary file 2 — Reporting Summary [file 41586_2023_6819_MOESM2_ESM.pdf]

nature portfolio

Corresponding author(s): Bing Ren, Joseph R. Ecker

Last updated by author(s): Nov 1, 2023

Reporting Summary

Nature Portfolio wishes to improve the reproducibility of the work that we publish. This form provides structure for consistency and transparency in reporting. For further information on Nature Portfolio policies, see our Editorial Policies and the Editorial Policy Checklist.

Statistics

For all statistical analyses, confirm that the following items are present in the figure legend, table legend, main text, or Methods section.

n/a	Confirmed	
☐	☒	The exact sample size (*n*) for each experimental group/condition, given as a discrete number and unit of measurement
☒	☐	A statement on whether measurements were taken from distinct samples or whether the same sample was measured repeatedly
☐	☒	The statistical test(s) used AND whether they are one- or two-sided<br>*Only common tests should be described solely by name; describe more complex techniques in the Methods section.*
☒	☐	A description of all covariates tested
☐	☒	A description of any assumptions or corrections, such as tests of normality and adjustment for multiple comparisons
☐	☒	A full description of the statistical parameters including central tendency (e.g. means) or other basic estimates (e.g. regression coefficient) AND variation (e.g. standard deviation) or associated estimates of uncertainty (e.g. confidence intervals)
☐	☒	For null hypothesis testing, the test statistic (e.g. *F*, *t*, *r*) with confidence intervals, effect sizes, degrees of freedom and *P* value noted<br>*Give P values as exact values whenever suitable.*
☒	☐	For Bayesian analysis, information on the choice of priors and Markov chain Monte Carlo settings
☒	☐	For hierarchical and complex designs, identification of the appropriate level for tests and full reporting of outcomes
☐	☒	Estimates of effect sizes (e.g. Cohen's *d*, Pearson's *r*), indicating how they were calculated

Our web collection on statistics for biologists contains articles on many of the points above.

Software and code

Policy information about availability of computer code

Data collection	No software was used for data collection.
Data analysis	cellranger-arc v2.0.0, cellranger-atac v2.0.0, cellranger v6.1.2, MACS2, YAP v1.6.8, cutadapt, v2.10, bismark v0.20, bowtie2 v2.3, allcools v1.0.8, methylpy, scHiCluster, cooltools v0.5.1, liftOver, HOMER, GSEApy, edgeR v3.36.0, FIMO v5.5.3, wigToBigWig, bigWigAvgOverBed, bedtools multicov, Seurat v4, DoubletFinder v2.0.3, Harmony v0.1.0, ABC: run.neighborhoods.py & predict.py , Basenji v0.6

For manuscripts utilizing custom algorithms or software that are central to the research but not yet described in published literature, software must be made available to editors and reviewers. We strongly encourage code deposition in a community repository (e.g. GitHub). See the Nature Portfolio guidelines for submitting code & software for further information.

Data

Policy information about availability of data

All manuscripts must include a data availability statement. This statement should provide the following information, where applicable:

- Accession codes, unique identifiers, or web links for publicly available datasets
- A description of any restrictions on data availability
- For clinical datasets or third party data, please ensure that the statement adheres to our policy

Data produced in this study are available in the NCBI Gene Expression Omnibus (GEO) under accession number GSE229169 for 10x multiome, GSE240297 for sn-m3C-seq and GSE246760 for Droplet Paired-Tag. Data is uploaded for viewing on the WashU Comparative Epigenome Browser data hub: https://

epigenome.wustl.edu/BrainComparativeEpigenome/. Reference genomes used are hg38, mm10, Mmul_10, cj1700_1.1.

Research involving human participants, their data, or biological material

Policy information about studies with human participants or human data. See also policy information about sex, gender (identity/presentation), and sexual orientation and race, ethnicity and racism.

Reporting on sex and gender	Human subjects were male.
Reporting on race, ethnicity, or other socially relevant groupings	*Please specify the socially constructed or socially relevant categorization variable(s) used in your manuscript and explain why they were used. Please note that such variables should not be used as proxies for other socially constructed/relevant variables (for example, race or ethnicity should not be used as a proxy for socioeconomic status).*<br>*Provide clear definitions of the relevant terms used, how they were provided (by the participants/respondents, the researchers, or third parties), and the method(s) used to classify people into the different categories (e.g. self-report, census or administrative data, social media data, etc.)*<br>*Please provide details about how you controlled for confounding variables in your analyses.*
Population characteristics	Donor ID, Sex, Age, PMI, hemisphere, Cause of Death<br>H19.30.001, Male, 42, 8h, right, suicide<br>H19.30.002, Male, 29, 7.5h, right, pulmonary embolism<br>H19.30.004, Male, 58, 12h, right, witnessed cardiac arrest
Recruitment	Postmortem donors with no known neuropsychiatric or neurological conditions between ages 18 and 68 were considered for inclusion.
Ethics oversight	Permission was obtained from decedent next-of-kin. Postmortem tissue collection was performed in accordance with the provisions of the United States Uniform Anatomical Gift Act of 2006 described in the California Health and Safety Code section 7150 (effective 1/1/2008) and other applicable state and federal laws and regulations. The Western Institutional Review Board reviewed tissue collection processes and determined that they did not constitute human subjects research requiring institutional review board (IRB) review.

Note that full information on the approval of the study protocol must also be provided in the manuscript.

Field-specific reporting

Please select the one below that is the best fit for your research. If you are not sure, read the appropriate sections before making your selection.

☒ Life sciences ☐ Behavioural & social sciences ☐ Ecological, evolutionary & environmental sciences

For a reference copy of the document with all sections, see nature.com/documents/nr-reporting-summary-flat.pdf

Life sciences study design

All studies must disclose on these points even when the disclosure is negative.

Sample size	Sample size was not predetermined, and were limited to available samples. Number of subjects were n = 3 for human, n = 3 for macaque, n = 3 for marmoset, and n = 8 mice.
Data exclusions	Low quality nuclei were removed from datasets. After predicted doublets were removed we used quality metrics to further remove low quality cells. For 10x multiome nuclei were required to have ≥1000 ATAC fragments and ≥500 genes detected. For sn-m3c-seq nuclei were required to have overall mCCC level < 0.05, overall mCH level < 0.2, overall mCG level < 0.5, total final reads > 500,000, and < 10,000,000, Bismarck mapping rate > 0.5. For Droplet Paired-Tag nuclei were required to have ≥200 genes detected.
Replication	Sample quality metrics, data quality metrics, and cell type proportions were assessed for each individual sample and displayed high reproducibility, n = 3 for primates, n = 4 for mouse. To obtain adequate sample sizes for downstream analysis, data across samples were subsequently combined for each cell type within each species individually.
Randomization	All samples were effectively controls, therefore randomization was not used and all samples were included in the same experimental group.
Blinding	Researchers used samples that were labeled with IDs and no identifying donor information within species, however researches were not blind to the species for each sample to exercise appropriate safety protocols.

Reporting for specific materials, systems and methods

We require information from authors about some types of materials, experimental systems and methods used in many studies. Here, indicate whether each material, system or method listed is relevant to your study. If you are not sure if a list item applies to your research, read the appropriate section before selecting a response.

Materials & experimental systems

n/a	Involved in the study
☐	☒ Antibodies
☒	☐ Eukaryotic cell lines
☒	☐ Palaeontology and archaeology
☐	☒ Animals and other organisms
☒	☐ Clinical data
☒	☐ Dual use research of concern
☒	☐ Plants

Methods

n/a	Involved in the study
☒	☐ ChIP-seq
☒	☐ Flow cytometry
☒	☐ MRI-based neuroimaging

Antibodies

Antibodies used	anti-H3K27ac (Abcam, ab4729), anti-NeuN antibody (MAB377X, Millipore)
Validation	ab4729 validated by manufacturer for ICC/IF, WB, IHC-P, ChIP, PepArr. Validated for droplet paired-tag signal-to-noise through transcription start site enrichment analysis. MAB377X is validated by manufacturer for IHC and reactivity in human, mouse, and rat.

Animals and other research organisms

Policy information about studies involving animals; ARRIVE guidelines recommended for reporting animal research, and Sex and Gender in Research

Laboratory animals	3 macaque donors (6 y.o. male Macaca mulatta, 6 y.o. male Macaca mulatta, and 14 y.o. male Macaca fascicularis), 3 marmoset (Callithrix jacchus) donors (5 y.o. male, 4 y.o. male, and 6 y.o. female), and MOp from 8 P56 C57BL/6J male mice (Mus musculus). C57Bl/6J animals, purchased from Jackson Laboratories, were kept for up to 10 days in the Salk animal barrier facility on a 12-hour dark/light cycle, under controlled temperature (between 20-22 degrees Celsius) and food ad-libitum.
Wild animals	No wild animals were used in this study
Reporting on sex	3 male macaque, 2 male and 1 female marmoset, 8 male mice
Field-collected samples	No field collected animals were used in this study
Ethics oversight	Mouse experiments were approved by the SALK Institute Animal Care and Use Committee under protocol number 18-00006. Marmoset experiments were approved by and in accordance with the Massachusetts Institute of Technology IACUC protocol number 05170520. Macaque experiment protocols were approved by the University of Washington Institutional Animal care and Use Committee.

Note that full information on the approval of the study protocol must also be provided in the manuscript.

Plants

Seed stocks	*Report on the source of all seed stocks or other plant material used. If applicable, state the seed stock centre and catalogue number. If plant specimens were collected from the field, describe the collection location, date and sampling procedures.*
Novel plant genotypes	*Describe the methods by which all novel plant genotypes were produced. This includes those generated by transgenic approaches, gene editing, chemical/radiation-based mutagenesis and hybridization. For transgenic lines, describe the transformation method, the number of independent lines analyzed and the generation upon which experiments were performed. For gene-edited lines, describe the editor used, the endogenous sequence targeted for editing, the targeting guide RNA sequence (if applicable) and how the editor was applied.*
Authentication	*Describe any authentication procedures for each seed stock used or novel genotype generated. Describe any experiments used to assess the effect of a mutation and, where applicable, how potential secondary effects (e.g. second site T-DNA insertions, mosiacism, off-target gene editing) were examined.*
